# Efficiency of Fulvestrant Monotherapy After CDK4/6 Inhibitor Exposure: Is This a Viable Choice?

**DOI:** 10.3390/cancers17050884

**Published:** 2025-03-04

**Authors:** Nanae Ogata, Brian G Barnett, Nicholas J. H. Sharp, Takeo Fujii, Toshiaki Iwase, Sandra E. Dunn, Naoto T. Ueno

**Affiliations:** 1 Translational and Clinical Research Program, University of Hawaiʻi Cancer Center, Honolulu, HI 96813, USA; 2 Cancer Biology Program, University of Hawaiʻi Cancer Center, Honolulu, HI 96813, USA; 3 Phoenix Molecular Designs Ltd., 1-8755 Ash St, Vancouver, BC V6P 6T3, Canada; 4 Women’s Malignancies Branch, Center for Cancer Research, National Cancer Institute, National Institutes of Health, Bethesda, MD 20892, USA

**Keywords:** breast cancer, fulvestrant, cyclin-dependent kinase 4 and 6 inhibitor (CDK4/6 inhibitor), antibody-drug conjugates (ADCs), protein kinase inhibitors, ribosomal protein S6 kinase 2 (RSK2)

## Abstract

Guidelines for the first-line treatment of Hormone Receptor-positive, HER2-negative advanced or recurrent breast cancer have shifted to combination therapies of a CDK4/6 inhibitor and endocrine therapy. However, determining an optimal subsequent therapy following CDK4/6 inhibitor progression remains challenging, especially for tumors lacking actionable mutations. Real-world data suggest that fulvestrant monotherapy is frequently selected in this post-CDK4/6 inhibitor setting. This review examines its therapeutic potential in this evolving landscape. A systematic literature search using PubMed and ClinicalTrials.gov identified 153 clinical trials published between 2017 and November 2024, from which ten studies met our strict inclusion criteria, focusing solely on fulvestrant monotherapy. These trials encompassed 1038 patients who had prior exposure to CDK4/6 inhibitors. The selected studies were categorized into three groups: monotherapy trials (EMERALD, SERENA-2, AMEERA-3, and ELAINE-1), combination therapy trials (CAPItello-291 and VERONICA), and CDK4/6 inhibitor rechallenge trials (post-MONARCH, PACE, PALMIRA, and MAINTAIN). The median progression-free survival for fulvestrant monotherapy was 3.18 months (range 1.9–5.3 months). Factors affecting the efficacy of fulvestrant monotherapy in second-line therapy include prior treatments, treatment duration, and genetic mutations. Given that the efficacy of fulvestrant was short-lived in the second or subsequent lines, participating in clinical trials is a vital option until a novel alternative treatment choice becomes available.

## Simple Summary

Amid the rise of new therapies for metastatic Hormone Receptor-positive, HER2-negative breast cancer, this review evaluates the role of fulvestrant monotherapy as a treatment following CDK4/6 inhibitors. Analyzing data from 10 clinical trials and 1038 patients, we found a median progression-free survival of 3.18 months. This review provides a comparative analysis of fulvestrant monotherapy alongside other second-line treatment options, highlighting its position in the evolving landscape of breast cancer therapies.

## 1. Introduction

The combination of cyclin-dependent kinase 4/6 (CDK4/6) inhibitors with endocrine therapy (ET) has become the first-line treatment for advanced or recurrent Hormone Receptor (HR)-positive, HER2-negative (HR+/HER2-) breast cancer since around 2017. In a post-CDK4/6 inhibitor setting, selecting an appropriate subsequent therapy remains challenging, particularly when tumors lack targetable gene mutations. According to real-world data (RWD), fulvestrant has been the most commonly chosen ET monotherapy [1]. Fulvestrant monotherapy is not listed under “Preferred Regimens” but is classified as an “Other Recommended Regimen” for first- and/or subsequent-line therapy in HR+/HER2- advanced breast cancer, according to current guidelines [2]. With over 20 years of clinical use, fulvestrant is recognized for its manageable side effect profile and is often employed as a comparator in clinical trials evaluating novel agents. However, its efficacy, particularly in a post-CDK4/6 inhibitor setting, still needs to be fully understood. In this review, we examined the therapeutic potential of fulvestrant monotherapy in the evolving treatment landscape, aiming to clarify its role in this challenging scenario.

In all trials, fulvestrant monotherapy was the control group, while target treatments involved new agents alone or combined with fulvestrant. The median of the individual median progression-free survival (mPFS) across these ten studies of fulvestrant monotherapy was 3.18 months. Only trials with those patients included 100% post-CDK4/6 inhibitors, except for the AMEERA-3 trial (the ratio of post-CDK4/6 inhibitors was 80%). Trials were excluded if the results of mPFS of the fulvestrant arm contained any other ET. However, we included the AMEERA3 and PALMIRA trials since about 90% of ETs were treated with fulvestrant.

**Figure 1:**
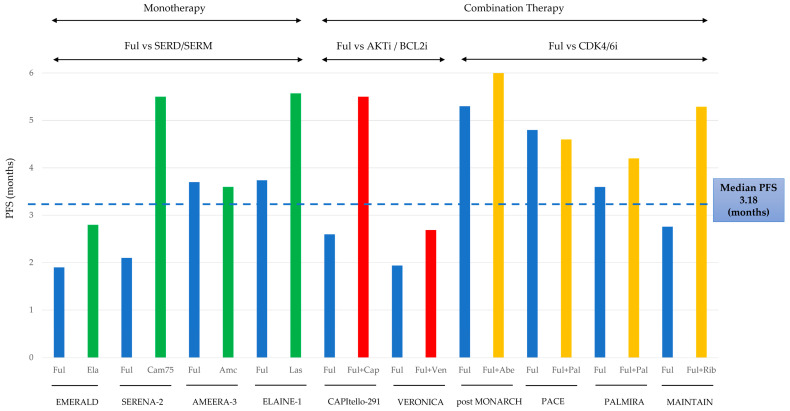
Median progression-free survival across 10 trials. Fulvestrant (blue bars) is the standard, novel SERMs and SERDs as monotherapies are shown in green, novel inhibitors in combination with fulvestrant in red, and CDK4/6 inhibitors in combination therapy in yellow. Ful, Fulvestrant; Ela, Elacestrant; Cam, Camizestrant; Amc, Amcenestrant; Las, Lasofoxifene; Cap, Capivasertib; Ven, Venetoclax; Abe, Abemaciclib; Pal, Palbociclib; Rib, Ribociclib; SERD, Selective Estrogen Receptor Degrader; SERM, Selective Estrogen Receptor Modulator; i, inhibitor.

Figure 1 presents the median progression-free survival (mPFS) of the treatment and control groups (fulvestrant monotherapy) across the 10 analyzed trials.

The first four trials—EMERALD, SERENA-2, AMEERA-3, and ELAINE-1—compared new-generation oral SERDs or SERM as monotherapy against fulvestrant monotherapy. CAPItello-291 and VERONICA evaluated novel combination therapies with fulvestrant, comparing them to placebo plus fulvestrant, effectively assessing the benefit of adding a new agent to fulvestrant monotherapy. The final four trials—post-MONARCH, PACE, PALMIRA, and MAINTAIN—investigated the reintroduction of a CDK4/6 inhibitor after prior CDK4/6 inhibitor treatment, comparing ET (including fulvestrant) plus a CDK4/6 inhibitor to fulvestrant monotherapy.

For study-specific details, the SERENA-2 trial group represents PFS data for the camizestrant 75 mg arm. The post-MONARCH trial data are based on investigator-assessed results from the primary analysis. In the PACE study, the treatment group includes patients receiving fulvestrant plus palbociclib. The PALMIRA trial data are reported with a median follow-up of 8.7 months.

## 2. Selection Criteria for Fulvestrant Monotherapy Analysis

To assess its efficacy specifically in a post-CDK4/6 inhibitor setting, we conducted a comprehensive literature search using PubMed and ClinicalTrials.gov, covering studies published between 2017 and November 2024. Our selection criteria were designed to ensure a focused analysis of fulvestrant monotherapy, independent of other ETs such as aromatase inhibitors (AIs). We prioritized Phase 2 and 3 clinical trials that reported outcomes specifically for fulvestrant monotherapy in patients with prior CDK4/6 inhibitor exposure. If a trial included multiple ET options as a control group, we only selected those that provided separate outcome data for fulvestrant, allowing for a clear evaluation of its efficacy without confounding from other ETs.

This search initially identified 153 clinical trials using the keywords “Fulvestrant, Breast Cancer” on PubMed. After applying our strict inclusion criteria, we narrowed the selection down to ten trials, encompassing 1038 patients (Table 1) [3,4,5,6,7,8,9,10,11,12,13,14,15,16]. Notably, we excluded trials where the reported median progression-free survival (mPFS) of the fulvestrant arm included patients treated with other ETs, ensuring a pure analysis of fulvestrant monotherapy. While we maintained a rigorous selection process, we included the AMEERA-3 and PALMIRA trials despite their control arms not being exclusively fulvestrant. In AMEERA-3, approximately 80% of patients in the control group had prior CDK4/6 inhibitor therapy, and in PALMIRA, nearly 90% of patients received fulvestrant as their ET. Given the high proportion of fulvestrant-treated patients in these studies, we determined that their inclusion would not significantly impact the validity of our analysis.

This refined selection process allows for a more precise assessment of fulvestrant monotherapy in a post-CDK4/6 inhibitor setting, ensuring that the reported outcomes are as representative and reliable as possible.

## 3. Background of Ten Trials, Including Fulvestrant Monotherapy Results

Below are the details of ten trials reporting the outcomes of fulvestrant monotherapy as a control group in a post-CDK4/6 inhibitor setting, including prior treatments, metastatic sites, genetic mutations, and racial distribution.

### 3.1. Monotherapy Studies (Green Bars in Figure 1)

The first four trials—EMERALD, SERENA-2, AMEERA-3, and ELAINE-1—were comparative studies evaluating new-generation estrogen receptor-targeting agents as monotherapy against fulvestrant monotherapy. Among these, elacestrant, camizestrant, and imlunestrant are emerging as next-generation selective estrogen receptor degraders (SERDs), while lasofoxifene is classified as a selective estrogen receptor modulator (SERM).

The EMERALD trial (NCT03778931) was a randomized, open-label, phase III trial, which focused on patients with prior CDK4/6 inhibitor treatment to evaluate the efficacy of elacestrant, a novel oral SERD [3]. Patients had received one to two lines of ET, required prior therapy with a CDK4/6 inhibitor, and had received no more than one line of chemotherapy. They were randomly assigned to receive either elacestrant 400 mg orally once daily (*n* = 239) or standard-of-care (SOC) treatment, with 165 receiving fulvestrant. Of these fulvestrant-treated patients, 117 (70.9%) had visceral metastases, and 83 (50.3%) had *ESR1* mutations.

The SERENA-2 trial (NCT04214288) was an open-label, randomized, phase 2 study evaluating the efficacy and safety of different doses of camizestrant compared to fulvestrant [4]. The fulvestrant group included 73 patients, of whom 37 had prior treatment with a CDK4/6 inhibitor, with a median PFS of 2.1 months (Table 1). Among them, 56 (77%) had received one prior line of ET (including adjuvant treatment); 14 (19%) had received two lines; and 3 (4%) had received three lines. Liver and/or lung metastases were present in 43 patients (59%), and *ESR1* mutations were detected in 35 (48%).

The AMEERA-3 trial (NCT04059484) was an open-label phase II study evaluating the efficacy and safety of amcenestrant in patients who had progressed in (neo)adjuvant or advanced settings after no more than two prior lines of ET [5]. Among the 147 patients assigned to the SOC group, 132 (89.9%) received fulvestrant monotherapy, 10 received AIs, and 5 received tamoxifen. Prior treatment with a CDK4/6 inhibitor was reported in 115 patients (78.2%). Visceral metastases were present in 94 patients (63.9%). Among the 140 patients tested for *ESR1* mutations, 55 (39.3%) had mutations.

The ELAINE 1 trial (NCT03781063) was an open-label, randomized phase II study comparing the novel SERM lasofoxifene with fulvestrant in patients with *ESR1* mutations who had previously received a CDK4/6 inhibitor [6]. The fulvestrant monotherapy group included 51 patients, 33 (64.7%) of whom had visceral metastases.

### 3.2. Combination with Novel Agents (Red Bars in Figure 1)

The remaining six studies compared fulvestrant monotherapy to a combination of fulvestrant and an agent [7,8,9,10,11,12]. The CAPItello-291 and the VERONICA trials evaluated novel combination therapies with fulvestrant, comparing them to placebo plus fulvestrant, effectively assessing the benefit of adding a new agent to fulvestrant monotherapy.

The CAPItello-291 trial (NCT04305496) was a phase III, randomized, double-blind study evaluating the efficacy and safety of capivasertib, an AKT inhibitor, in combination with fulvestrant versus fulvestrant alone [7,17]. Among the 353 patients in the fulvestrant group, 248 were post-CDK4/6 inhibitor treatment, with a median PFS of 2.6 months (Table 1). Although this includes all 353 patients in the fulvestrant group, the breakdown of the number of previous therapies for advanced breast cancer was as follows: 52 patients (14.7%) were treatment-naïve; 208 patients (58.9%) had received one prior line; 77 patients (21.8%) had received two lines; and 16 patients (4.5%) had received three lines. Additionally, 134 patients (38.0%) in the fulvestrant monotherapy group had mutations in the AKT pathway (*PIK3CA*, *AKT1*, or *PTEN*). There were 241 patients (68.3%) with visceral metastases, including 150 patients (42.5%) with liver metastases.

The VERONICA(NCT03584009) trial was a randomized phase II clinical trial evaluating veratoclax (a B-cell lymphoma 2 inhibitor) and fulvestrant in post-CDK4/6 inhibitor progression [8]. In the fulvestrant group, there were 52 patients. Regarding the line of prior metastatic breast cancer ET, 43 patients (82.7%) had received one line, while nine patients (17.3%) had received two lines. There were 43 patients (82.7%) with visceral metastasis. Mutations were detected in 46 patients, with *ESR1* mutations observed in 19 patients (41.3%), followed by *TP53* mutations in 16 patients (34.8%), and *PIK3CA* mutations in 14 patients (34.6%).

### 3.3. CDK4/6 Inhibitor Rechallenge Studies (Yellow Bars in Figure 1)

The following four trials have investigated the benefits of reusing various CDK inhibitors in patients with a prior history of ET plus CDK4/6 inhibitor combination therapy.

The post-MONARCH (NCT05169567) trial was a double-blind, randomized phase III study and enrolled patients with disease progression on previous CDK4/6 inhibitors plus an AI as an initial advanced disease therapy [9]. A total of 186 patients were enrolled in the placebo plus fulvestrant group, with visceral metastases observed in 109 patients (58.6%). ctDNA analysis was performed in 159 patients, revealing the most common mutations as follows: *ESR1* mutations in 81 patients (50.9%), *PIK3CA* mutations in 67 patients (42.1%), *PTEN* mutations in 16 patients (10.1%), and *AKT1* mutations in 9 patients (5.7%).

The PACE(NCT03147287) study was a randomized phase II trial and enrolled progressed disease patients with previous CDK4/6 inhibitors and an AI [10]. Among the 220 enrolled patients, 55 were randomly assigned to the fulvestrant monotherapy group. Visceral disease was observed in 29 patients (52.7%). Regarding the line of metastatic breast cancer therapy initiated in this study, 3 patients (5.5%) received it as first-line treatment, 42 patients (76.4%) as second-line treatment, and 10 patients (18.3%) as third-line or beyond. The most common genetic mutations were *ESR1* in 23 patients (47.9%), *PIK3CA* in 12 patients (25%), and *RB* in 6 patients (12.5%).

The PALMIRA(NCT03809988) trial was a randomized phase II trial. A total of 198 patients who had experienced disease progression on first-line palbociclib plus ET (either an AI or fulvestrant) were included [11]. Patients were randomly assigned to treatment, with ET selected based on prior therapy, choosing either letrozole or fulvestrant. Given that 89.9% of patients had received an AI as prior ET, it can be inferred that most patients in the ET group of this trial opted for fulvestrant. A total of 61.1% had visceral disease, and details on genetic mutations and racial distribution were not reported.

The MAINTAIN(NCT05207709) trial was a randomized phase II, double-blind, placebo-controlled trial in patients with progressed disease with ET plus CDK4/6 inhibitor [12]. A total of 59 patients were assigned to the placebo plus ET group, with 50 selecting fulvestrant and 9 selecting exemestane. The mPFS of 2.76 months, as shown in Table 1, represents data from the fulvestrant group, excluding exemestane; however, the overall PFS for the entire ET group was also 2.76 months. A total of 35 patients (59.3%) had visceral metastasis. Regarding previous lines of ET in a metastatic setting, 37 patients (62.7%) received one prior line, while 9 patients (15.3%) received two. In the fulvestrant group, 15 individuals had *ESR1* mutations, and 10 had *PIK3CA* mutations.

## 4. Efficacy of Fulvestrant Monotherapy in a Post-CDK4/6 Inhibitor Setting

Overall, calculating the mPFS across these ten clinical trials, the mPFS for patients treated with fulvestrant alone after CDK4/6 inhibitor therapy was 3.18 months (range: 1.9–5.3 months). Nur et al. reported a real-world (rw) mPFS of 3.9 months for fulvestrant monotherapy in 179 patients, while a second study observed a rw-PFS of 3.25 months in 70 of 839 patients treated with fulvestrant single-agent therapy, both of which closely align with our findings [1,18]. A three-month PFS in the post-CDK4/6 inhibitor treatment line raises whether this duration is reasonable. In the adjusted analysis from Martin et al., the mPFS for the post-CDK4/6 inhibitor cohorts receiving chemotherapy or everolimus was 3.71 and 3.32 months, respectively, which was not significantly different from fulvestrant. Continuation of CDK4/6 inhibitors (whether the same or a different CDK4/6 inhibitor was used) significantly improved rwPFS at 8.25 months compared to chemotherapy (HR 0.48, 95% CI 0.43–0.53, *p* < 0.0001). In first-line therapy, the most frequently chosen combination was palbociclib (88.2%) with letrozole (61.6%). After progression, the most common treatments were chemotherapy (29.7%), the combination of a CDK4/6 inhibitor with fulvestrant (19.1%), everolimus (11.7%), a CDK4/6 inhibitor with an AI (11.6%), and fulvestrant monotherapy (8.6%); however, this analysis is based on a retrospective evaluation of data from 2015 to 2020 [1].

An mPFS of just over three months raises important questions about the clinical relevance of fulvestrant monotherapy in a post-CDK4/6 inhibitor setting and which patients might benefit most from this approach. This outcome suggests that fulvestrant monotherapy provides a level of efficacy comparable to chemotherapy or everolimus but is inferior to rechallenging with a CDK4/6 inhibitor. Additionally, in cases where specific genetic mutations are identified, alternative treatments such as next-generation oral SERDs may offer greater benefit. While fulvestrant will remain an option for patients with HR+/HER2- metastatic breast cancer, particularly those without actionable mutations, multiple comorbidities, or concerns about adherence to targeted therapy, it is crucial to determine which clinical and biological factors can help identify patients who may derive the most benefit. In the following sections, we examine four key factors that may influence the efficacy of fulvestrant monotherapy.

## 5. Factors Influencing the Therapeutic Effect of Fulvestrant

### 5.1. Duration of Previous Treatment

In the subgroup analysis of the post-MONARCH study, patients were divided based on whether their previous treatment with ET and CDK4/6 inhibitors lasted 12 months or more [9]. For those with a treatment duration of 12 months or longer, the mPFS of fulvestrant monotherapy in the second line was 5.4 months, while it was 3.0 months for those treated for less than 12 months. These results suggest that a longer period of pretreatment may result in a longer treatment effect. In the EMERALD study (NCT03778931), which targeted only the ESR1-mutated subgroup, however, fulvestrant monotherapy showed minimal differences in mPFS across prior ET + CDK4/6i durations of ≥6, ≥12, and ≥18 months, with values of 1.87, 1.87, and 2.10 months, respectively [19]. A retrospective study by Lerner et al. involving 429 patients treated with fulvestrant monotherapy did not report the duration of prior treatments but found no significant difference in mPFS between 0-line, 1–2-line, and 3–4-line treatments (*p* = 0.125) [20]. In other words, the duration of prior treatment does not appear to be a key factor when selecting fulvestrant monotherapy.

### 5.2. Metastasis Site

In a previous retrospective study, the efficacy of fulvestrant varied by metastatic site, though not all patients had prior CDK4/6 inhibitor treatment [20]. Patients without visceral metastases had a median PFS of 6 months, compared to 5 months in those with visceral metastases (*p* = 0.042). Among patients with bone-only metastases, the median PFS was 7 months, whereas it was 5 months for those with lymph node or visceral metastases (*p* = 0.220). Notably, over 20% of patients without visceral metastases achieved a PFS greater than 30 months, suggesting fulvestrant may be particularly effective in this group.

In our selected ten trials, the two with the shortest mPFS—EMERALD (1.9 months) and VERONICA (1.94 months)—had the highest proportions of visceral metastases at 70.9% and 82.7%, respectively. In contrast, the two trials with the longest mPFS—post-MONARCH (5.3 months) and PACE (4.8 months)—had the lowest proportions of visceral metastases at 58.6% and 52.7%, respectively. These findings suggest that visceral metastases may influence PFS outcomes.

In the EMERALD trial, however, among patients with ESR1-mutated tumors and prior ET + CDK4/6 inhibitor therapy for ≥12 months, the mPFS for SOC (90% fulvestrant) was consistently low (1.9 months), regardless of metastatic site—1.9 months for bone metastases, 1.9 months for liver and/or lung metastases, 1.9 months for fewer than three metastatic sites, and 1.8 months for three or more sites [19]. This suggests that while metastatic site distribution may contribute to PFS differences across studies, it is not a reliable predictor of fulvestrant monotherapy efficacy.

### 5.3. Impact of ESR1 Mutation

The PACE trial demonstrated the efficacy of fulvestrant monotherapy, with an mPFS of 4.6 months overall, 7.6 months in the *ESR1* wild-type group, and 3.3 months in the *ESR1*-mutant group. Similarly, the EMERALD study reported an mPFS of 3.7 months in the ESR1 wild-type group and 1.9 months in the mutant group, showing differing outcomes based on ESR1 mutations [3]. In the same study, in the ESR1-mutated subgroup with a prior ET + CDK4/6i treatment of ≥12 months, elacestrant achieved an mPFS of 8.6 months, whereas fulvestrant achieved 2.1 months [19]. Although the proportion of patients post-CDK4/6 inhibitor is limited to 60%, the EMBER-3 trial (Dec 2024, NCT04975308) evaluated another novel SERD, imlunestrant, and similar results were observed. In *ESR1*-mutated patients, the mPFS was 5.5 months with imlunestrant vs. 3.8 months with SOC (~90% fulvestrant, *p* < 0.001) [21]. Based on these results, in patients with ESR1 mutations, new SERDs may offer better treatment efficacy, and fulvestrant monotherapy is not recommended; new SERDs should be considered as an alternative.

### 5.4. Kinome Reprograming

In the PACE trial, the mPFS for fulvestrant monotherapy was 7.6 months in the *PI3KCA* wild-type group, compared to 2.0 months in the mutation group. For the wild-type *RB1* group, the mPFS was 5.7 months compared to 1.9 months in the mutation group. In patients without mutations, fulvestrant monotherapy is associated with longer PFS. As a case of combining with targeted therapy, in the CAPItello-291 trial, the mPFS was 7.3 months for the AKT inhibitor capivasertib plus fulvestrant, compared to 3.1 months for the placebo plus fulvestrant in the AKT pathway-altered population (*PIK3CA*, *AKT1*, or *PTEN*) [7]. The BYLieve trial targeted patients with prior CDK4/6 inhibitor treatment, and *PIK3CA* mutations reported an mPFS of 7.3 months with alpelisib plus or minus fulvestrant at a median follow-up of 11.7 months [22]. According to these studies, appropriate targeted therapies are expected to result in longer PFS, even in patients with mutations.

## 6. Situations Where Fulvestrant Monotherapy May Be Beneficial

It is crucial to consider the potential for treatment resistance when making treatment decisions. Since various genetic mutations can develop during ET, conducting genomic testing is essential, especially in patients with a history of extended treatment with CDK 4/6 inhibitors who are then experiencing disease progression. This strategy can help tailor treatment approaches for better outcomes. The choice of second-line therapy after the CDK4/6 inhibitor at present depends mainly on the presence of specific mutations. As of winter 2024, a PARP inhibitor is recommended for a germline *BRCA* mutation [23,24]. If an *ESR1* mutation develops during treatment, an elacestrant should be used. For *PIK3CA* mutations, combination therapy with alpelisib and fulvestrant or triple therapy with inavolisib, palbociclib, and fulvestrant are preferred [25]. If *PIK3CA*, *AKT*, or *PTEN* alterations are present, capivasertib combined with fulvestrant should be selected. As a prospect, in the absence of PIK3CA/AKT/PTEN pathway mutations, new targets are being explored due to the link between MAPK and PI3K pathways and CDK4/6i resistance, like RSK2, as potential targets for overcoming this resistance, and further clinical research is anticipated.

For patients without specific genetic mutations or when mutations cannot be evaluated, second-line options include continuing ET with CDK4/6 inhibitors with possible drug adjustments, switching to everolimus plus fulvestrant or exemestane, or using fulvestrant monotherapy [2,26,27,28,29]. In recent years, multiple ADCs have been approved, expanding the available treatment options [30,31]. However, their use requires careful consideration of side effects and financial burden. As these agents are classified as chemotherapy-like drugs, they are generally considered after exhausting endocrine therapy options whenever possible. Clinical trials are also reasonable options. When no clinical trial is available, fulvestrant monotherapy is appropriate for tumors without gene mutations. Further, even in the presence of mutations, some patients may not be eligible for combination therapies due to concerns about severe side effects or financial constraints.

## 7. Conclusions

Our findings indicate that fulvestrant monotherapy following CDK4/6 inhibitors results in a three-month mPFS, which is quite short but not surprising. If no oncogenic mutations are identified and the duration of prior treatment has been extended, fulvestrant alone may still offer a slightly longer benefit. Additionally, fulvestrant monotherapy remains a viable option when side effects, limitations in genetic mutation testing, or affordability restrict access to other combination therapies or clinical trials.

However, it should be emphasized that given the limited clinical benefit of fulvestrant, post-CDK4/6 single-agent endocrine therapy represents a significant area of unmet clinical need. Over the past decade, new targeted inhibitors have transformed HR+ HER2-advanced breast cancer treatment, markedly extending patient outcomes. The ongoing development of innovative agents provides hope for further survival improvements in the future. In conclusion, seeking and participating in clinical trials are the most critical options for post-CDK4/6 inhibitor usage in HR+ HER2- metastatic breast cancer.

## Figures and Tables

**Table 1:** Ten studies including fulvestrant monotherapy arms post-CDK4/6 inhibitor therapy.

	Trials Name (NCT ID) [Reference]	Phase	Investigatio- nal Agents	Category	Placebo Agents	FUL (n)	Prior ET Lines	Post-CDK 4/6i(%)	mPFS Months (Range)	Events (n)	Hazard Ratio	HR-95%CI	*p*-Value
1	EMERALD (NCT03778931) [3]	R-III	Elacestrant	SERD	Ful	165	1, 2	100	1.9 (NA)	109	0.68	0.52–0.90	0.049
2	SERENA-2 (NCT04214288) [4]	R-II	Camizestrant	SERD	Ful	37	1+	100	2.1 (1.9–3.7)	33	0.49	0.31–0.75 **	NA
3	AMEERA-3 (NCT04059484) [5]	R-II	Amcenestrant	SERD	Ful/AIs/TAM	132	0, 1, 2	78.2	3.7 (2.0–4.9) *	95	1.051	0.789–1.4	0.6437
4	ELAINE-1 (NCT03781063) [6]	R-II	Lasofoxifene	SERM	Ful	51	1	100	3.74 (2.7–5.6)	NA	0.699	0.434–1.125	0.138
5	CAPItello-291 (NCT04305496) [7]	R-III	Capivasertib	AKTi	Ful	248	1	100	2.6 (2.0–3.5)	216	0.59	0.48–0.72	NA
6	VERONICA (NCT03584009) [8]	R-II	Venetoclax	BCL2i	Ful	52	1	100	1.94 (*1–7*)	NA	0.94	0.61–1.45	0.7853
7	post MONARCH (NCT05169567) [9]	R-III	Abemaciclib	CDK4/6i	Ful	186	1+	100	5.3 (3.7–5.6)	NA	0.73	0.57–0.95	0.02
8	PACE (NCT03147287) [10]	R-II	Palbociclib	CDK4/6i	Ful	55	1	100	4.8 (2.1–8.2)	34	1.11	0.79–1.55 **	0.62
9	PALMIRA (NCT03809988) [11]	R-II	Palbociclib	CDK4/6i	Ful or LET	62 *	1	100	3.6 (2.7–4.2) *	NA	0.8	0.6–1.1	0.206
10	MAINTAIN (NCT05207709) [12]	R-II	Ribociclib	CDK4/6i	Ful or EXE	50	1	100	2.76 (2.66–3.25)	47	0.6	0.39–0.94	NA

* The data represent the ET group, which includes fulvestrant but is not limited to fulvestrant monotherapy. ** These data are 90% CI. The SERENA-2 trial adopted data comparing camizestrant 75 mg. Italicized values are estimated from Kaplan–Meier curves. R, Randomized; SERD, Selective Estrogen Receptor Degrader; SERM, Selective Estrogen Receptor Modulator; i, inhibitor; FUL, Fulvestrant; AIs, Aromatase inhibitors; TAM, Tamoxifen; LET, Letrozole; EXE, Exemestane.

## Data Availability

The datasets used and/or analyzed during the current study are available from the corresponding author upon reasonable request.
